# Peptides and Reactive Oxygen Species Regulate Root Development

**DOI:** 10.3390/ijms26072995

**Published:** 2025-03-25

**Authors:** Larisa Ivanovna Fedoreyeva, Neonila Vasilievna Kononenko

**Affiliations:** All-Russia Research Institute of Agricultural Biotechnology, Timiryazevskaya 42, 127550 Moscow, Russia; nilava@mail.ru

**Keywords:** root development, peptide, ROS

## Abstract

Like phytohormones, peptide hormones participate in many cellular processes, participate in intercellular communications, and are involved in signal transmission. The system of intercellular communications based on peptide–receptor interactions plays a critical role in the development and functioning of plants. One of the most important molecules are reactive oxygen species (ROS). ROS participate in signaling processes and intercellular communications, including the development of the root system. ROS are recognized as active regulators of cell division and differentiation, which depend on the oxidation–reduction balance. The stem cell niche and the size of the root meristem are maintained by the intercellular interactions and signaling networks of peptide hormone and ROS. Therefore, peptides and ROS can interact with each other both directly and indirectly and function as regulators of cellular processes. Peptides and ROS regulate cell division and stem cell differentiation through a negative feedback mechanism. In this review, we focused on the molecular mechanisms regulating the development of the main root, lateral roots, and nodules, in which peptides and ROS participate.

## 1. Introduction

**Figure 1:**
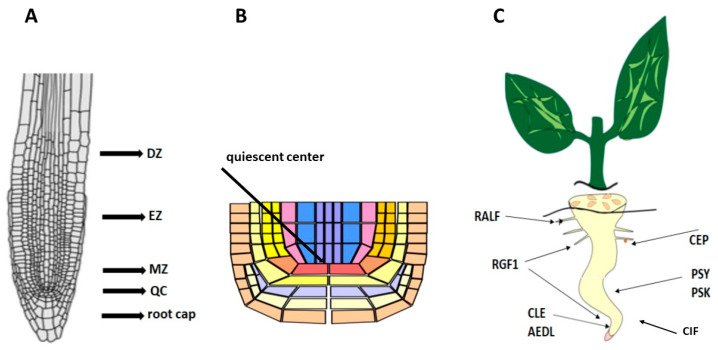
(**A**) Root zones. QC—quiescent center, MZ—meristematic zone, EZ—elongation zone, DZ—differential zone. (**B**) Quiescent center. (**C**) Localization of peptides in root zones.

The root is the main organ responsible for the absorption of nutrients. In a longitudinal section through the apical part of the root, four characteristic zones are distinguished: the root cap, the apical meristem, the elongation zone, the zone of cell differentiation, and the formation of permanent tissues (Figure 1A).

The specialization of the root as an organ of absorption, accumulation, conduction of substances, and anchoring of the plant in the substrate is associated, first of all, with an increase in the surface in direct contact with the soil. This is achieved by its branching, increasing the power of the root system, and the formation of many root hairs, which significantly increase the absorbent surface of the roots. During the development of the rudiment of the lateral root, a local growth of the meristem in thickness occurs. A further increase in its size is accompanied by the isolation of a group of small, actively dividing cells of the future apical meristem at the end of the root. Continuous growth of the root system is possible only in the presence of a pool of stem cells, which replenishes and restores the pool of meristem cells. A very important feature of the root apical meristem is that the initial cells themselves, under normal conditions, divide very rarely, forming a quiescent center (QC) [1], consisting of mitotically inactive stem cells that can replace root cells after their damage [2,3]. In most dicots, the initial cells are arranged in three layers. The derivatives of the upper layer subsequently form the central cylinder, the cells of the middle layer give rise to the primary cortex, and those of the lower layer give rise to the cells of the cap and rhizoderm. Root growth occurs due to controlled cell division in the meristematic zone. In the meristems, stem cells receive maintenance signals from QC cells [1]. Stem cell division results in the formation of rapidly dividing cells that migrate out of the meristem and then differentiate into specialized cell types or tissues [4]. After division, most cells increase in size in the elongation zone and mature in the differentiation zone. The size of these developmental zones is determined by internal and external signals. The root system architecture is determined by complex interactions between internal root development and external environmental signals. These feedback mechanisms are mediated through signaling molecules such as ROS, transcription factors, and secreted peptides [5].

Peptide hormones play key roles in many physiological processes by coordinating developmental and environmental signals between different cells (Figure 1C) [6,7,8]. Peptide hormones are recognized by their receptors, which transmit signals to downstream targets and interact with several pathways to fine-tune plant growth. Functional studies on the mechanisms of peptide action in roots are scarce. As numerous studies have shown, reactive oxygen species (ROS) are active participants in all cellular processes regulating plant development. In this review, we focus on recent advances in understanding the mechanisms of regulation of plant root development by peptide hormones, and their relationship with receptors and ROS.

## 2. ROS and Role of ROS in Root Development

ROS are a product of redox reactions that occur in all cells during plant life [9]. In plant cells, redox homeostasis is a normal state [10]. Under normal conditions, ROS production and levels are safe for normal cellular function, including proliferation, differentiation, signaling, and intercellular communication. The interplay between ROS and redox changes/regulation in cells is commonly referred to as redox biology and is thought to play an important role in ROS cellular signaling and metabolic regulation [11,12,13,14]. Since the membrane acts as a barrier to redox levels, each intracellular compartment can contain its own redox state, corresponding to its steady-state ROS levels, contributing to the formation of specific intracellular ROS signatures during abiotic stress [15]. The level of ROS is strictly controlled in space and time as a result of changes in the composition of ROS production and its detoxification [16]. At low levels in the cell, ROS act as positive signaling molecules, actively participating in many biologically important processes. At elevated concentrations, ROS can act as highly toxic molecules. The accumulation of ROS in a cell depends on many parameters. The complex process of plant adaptation includes a whole range of mechanisms of interaction between oxidants and antioxidants, capable of flexibly changing redox signals [17].

ROS are divided into two groups: ROS generated as a result of metabolic dysfunction (metabolic ROS) and ROS generated for signaling purposes as part of a signaling network in response to abiotic stress (signaling ROS). Metabolic ROS can counteract the effects of stress by directly altering the redox state of rate-limiting enzymes and controlling cellular metabolic fluxes, thereby altering various metabolic reactions [18]. In addition, ROS-induced redox modifications can affect transcription and translation by altering the functions of key regulatory proteins [11,12].

ROS metabolism is a very complex multicomponent system. Changing one of its elements may not lead to the desired effect due to the variety of enzyme isoforms. ROS metabolism can be influenced by environmental factors, such as bright light, ion imbalance, and damage. An increase in ROS levels is often observed under the influence of all these factors. It has been noted that disruption of the mitochondrial complex, which led to oxidative stress in the mitochondria, affected ROS metabolism in the apoplast [18]. ROS generation in mitochondria or chloroplasts during metabolic fluctuations can cause ROS production in the extracellular space.

Recent studies demonstrate the ever-increasing role of reactive oxygen species (ROS) as signaling molecules regulating development and various physiological processes in plants [19,20]. ROS include numerous small molecules such as singlet oxygen, as well as superoxide (O_2_^•−^), which oxidize lipids, proteins, and DNA. Hydrogen peroxide (H_2_O_2_), being a relatively stable molecule capable of penetrating cell membranes, is considered a key ROS involved in intercellular communication [21]. ROS interact with many other signaling molecules, which leads to a variety of cellular responses. Modern transcriptomic methods have identified important stress-sensitive signaling molecules such as mitogen-activated protein kinase (MAPK) and phosphatase, which play a role in ROS metabolism [22]. MAPK signaling networks that recognize stress stimuli through cell surface receptors and sensors are transduced [23]. Ca^2+^ ions are also actively involved in intracellular and extracellular signaling networks [24]. There is a two-way interaction between Ca^2+^ and ROS: Ca^2+^ is required for ROS production, and ROS, in turn, regulate cellular Ca^2+^ levels [25,26]. Plant cells exposed to stress factors transmit signals through redox-sensitive proteins through reversible oxidation–reduction reactions [27]. In addition, thiols, iron- and sulfur-containing compounds, and proteins are targets of ROS [28].

ROS are key regulators of subcellular, cellular, and systemic signals. They function in plants as an integral part of a variety of hormonal, physiological, and developmental processes, and play a critical role in defense and adaptive responses to various biotic and abiotic conditions [29]. ROS signals are integrated through intracellular networks and coordinate plant physiological responses [30]. ROS mediate a rapid, whole-plant systemic signal called the “ROS wave”, required to induce a state of systemic acquired acclimation or a systemic wound response. The authors show that the wound-induced systemic ROS surge in *Arabidopsis thaliana* is accompanied by a rapid systemic “redox surge”, which in turn can induce transcriptional, metabolic, and proteomic changes that lead to acclimation and/or systemic wound responses [31]. Intercellular communication and long-range signaling play an important role in plant responses to extreme environmental conditions. ROS is involved in organelle-to-organelle and cell-to-cell signaling. Signal propagation was accompanied by ROS accumulation in the extracellular spaces between cells and was inhibited by the suppression of ROS accumulation at sites distant from the initiation site [28,32,33].

Thus, ROS are key signaling molecules that allow cells to respond quickly to various stimuli. In plants, ROS play a critical role in recognizing abiotic and biotic stresses, integrating various environmental signals and activating stress response networks, thereby promoting the development of defense mechanisms and increasing plant resilience.

There is increasing evidence that redox regulation can significantly affect root development. Reactive oxygen species (ROS) act as key signaling molecules by interacting with antioxidants such as ascorbic acid (ASC) and glutathione (GSH) [34]. Studies have shown that ASC oxidase plays a role in root cell maintenance. Mutants of the rml1 gene, which is involved in the synthesis of GSH, an important molecule for cellular redox homeostasis and meristem maintenance, and other mutants have been widely used to demonstrate the roles of ROS, ASC, and GSH in root meristem function [35,36]. *rml1* mutants block root cell division, which prevents the formation of an active meristem; exogenous GSH can correct the *rml1*-associated defect. Furthermore, impaired GSH function inhibits cell division during the G1 to S transition [36]. A mutant of *UPB1*, a bHLH transcription factor that regulates the transition from cell proliferation to differentiation in the elongation zone, exhibits a delay in the differentiation process [37]. These results highlight the importance of redox regulation in maintaining the root meristem structure. This is further supported by the fact that O_2_^•−^ and H_2_O_2_ are unevenly distributed in roots, which affects their growth and differentiation [38]; O_2_^•−^ and H_2_O_2_ have different functions and accumulate in the root tips, with the former mainly located in the meristematic zone and the latter in the differentiation zone, participating in growth restriction and root hair formation [38].

## 3. Glutathione and Role of Glutathione in Root Development

The tripeptide glutathione (γ-glutamylcysteinylglycine) is one of the most abundant low-molecular compounds in plant tissues. GSH is a unique peptide that forms a peptide bond between the amino group of cysteine and the carboxyl group of the side chain of glutamic acid. Since GSH performs many functions in cells, it is found in almost all cellular compartments: the cytosol, endoplasmic reticulum, vacuoles, and mitochondria [39]. GSH is a water-soluble antioxidant that is involved in the scavenging of reactive oxygen species via the glutathione–ascorbic acid cycle and acts as an electron donor for glutathione peroxidase (GPX). GSH is the primary form in which sulfur is stored, and reduced sulfur is transported over long distances. GSH is capable of binding to heavy metals. It acts as a precursor of phytochelatin, which is a polymer of glutathione [40,41]. In addition, GSH is involved in the process of detoxification of harmful substances [5], together with glutathione transferase (GST) [42], which catalyzes the formation of covalent bonds between the sulfur atom in the cysteine residue of glutathione and electrophilic compounds [43]. GST catalyzes the reduction of H_2_O_2_ due to GSH, resulting in the formation of the oxidized form GSSG [44]. GSH is involved in signaling through the formation of disulfide bonds with various proteins. Changes in the antioxidant activity of glutathione are accompanied by changes in its participation in cellular signaling pathways and interaction with various glutathione-dependent enzymes [45].

**Figure 2:**
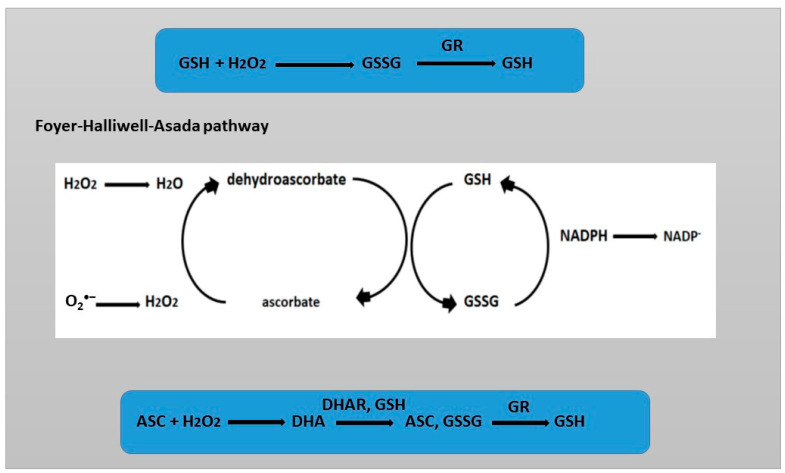
Redox reactions of GSH and ASC. Foyer–Halliwell–Asads pathway.

GSH contains a cysteine thiol group and functions as a proton donor (Figure 2). As a result, GSH can undergo reversible oxidation, leading to the formation of glutathione disulfide (GSSG), which is then reduced by glutathione reductase (GR). Under control conditions, when there is no stress, the GSH/GSSG ratio is 20:1. However, in the presence of stress, this ratio changes. Comparison of the reduced and oxidized forms of glutathione (GSH/GSSG) allows us to assess the level of oxidative stress, which is one of the key parameters stabilizing the cell state. The oxidation–reduction potential depends on the concentration of GSH. Even if the GSH/GSSG ratio remains unchanged, but the GSH concentration increases, this will lead to a decrease in the potential [46]. A distinctive feature of GSH from other primary and secondary metabolites, which can also react with ROS, is the rapid reduction of its oxidized form [47]. GSH in the cell is contained in millimolar concentrations. Such high levels of GSH and the rapid rate of reduction of its oxidized form GSSG provide it with an indispensable role in the redox homeostasis of the cell. GSH in the cell is contained in millimolar concentrations. Such high levels of GSH and the rapid rate of reduction of its oxidized form GSSG provide it with an indispensable role in the redox homeostasis of the cell. The reduction of the oxidized form of GSSG is carried out by the enzyme glutathione reductase (GR) [48].

**Figure 3:**
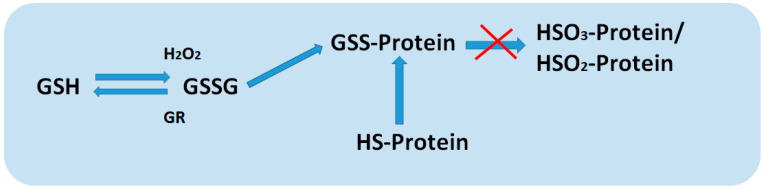
Mechanism of protection of protein thiol groups from oxidation.

Glutathione is able to protect proteins from denaturation as a result of the oxidation of cysteine residues during exposure to stressors (Figure 3). The protection of thiol groups in proteins occurs during the formation of a disulfide bond between GSH and the protein, which is temporary and is restored by GR. In addition, these short-lived disulfide bonds that arise between GSH and proteins can be a pair for the transfer of information [45].

The central role of GSH in antioxidant defense is due to its ability to reduce another potent water-soluble antioxidant, ascorbic acid, through the ascorbate–glutathione cycle [49]. ROS such as superoxide ion and hydroxyl are able to directly oxidize ASC and GSH at a high rate. The importance of ASC and GSH is determined by the fact that there are specific enzymes that carry out catalytic conversions into oxidized, which are stable, and reduced forms [39]. The alternation of redox states of GSH helps maintain the redox balance of ASC or glutaredoxin/thioredoxin [39,49,50]. Together with its oxidized form (GSSG), glutathione maintains the redox balance in cellular compartments. In addition, the reduced form of GSH, as an electron donor, is capable of reducing dehydroascorbate as one of the stages of the ASC—GSH cycle [49].

Thus, ASC and GSH, interacting together, are part of a very complex antioxidant system in plants [51]. However, these two compounds are not interchangeable and can perform different functions [52,53]. By maintaining a pool of reduced ASC, GSH is involved in the regulation of the cell cycle [54,55,56,57] and cell elongation [58]. Work with GSH-deficient *Arabidopsis* mutants has shown that glutathione performs critical functions in embryo and meristem development [47,59].

As a result of various negative environmental influences, plant development processes slow down [60]. Using *Arabidopsis* mutants as an example, it has been shown that GSH is involved in the regulation of plant growth and adaptation to biotic and abiotic environmental conditions [61,62]. A significant increase in the redox potential leads to damage to compartments sensitive to redox effects, which is ultimately accompanied by growth arrest and/or even death [63]. It is known that a high oxidative status is maintained in the root dormancy center responsible for root cell elongation [64,65]. However, in addition to partial cell death caused by high redox potential, it also affects processes associated with the fate of stem cells [66]. Genetic studies using mutants deficient in GSH and its biosynthesis inhibitor, buthionine sulfoximine, have shown that GSH plays an important role in the development of the primary root and lateral root (LR) [59,67]. One possible mechanism of GSH action on the development of these roots is to suppress the expression of PIN-forming proteins, which in turn regulates auxin transport [59,67]. The PIN4 protein is located in the plasma membrane of the quiescent center and surrounding cells, which may contribute to a decrease in auxin concentration in this center [68,69]. The proper functioning of PIN proteins is necessary for the maintenance of the root terminal meristem [69] and the formation of the lateral root primordium [70].

**Figure 4:**
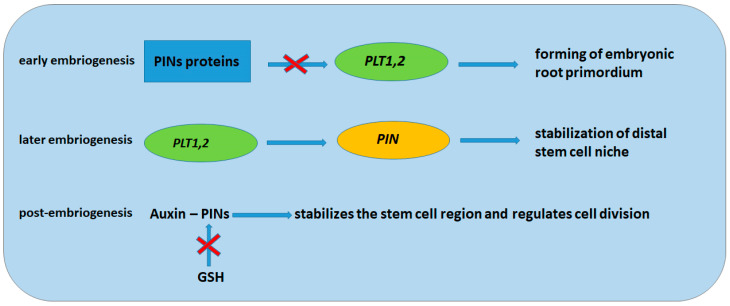
Mechanisms of embryonic root primordium formation and stabilization.

Figure 4 shows the mechanisms of regulation of the formation and stabilization of the embryonic root primordium with the participation of PIN proteins and the transcription factor PLETHORA (PLT) at different stages of embryogenesis. At an early stage, PIN proteins inhibit *PLT* expression in the basal region of the embryo and initiate the formation of the root primordium. At a later stage of embryogenesis, *PLT* genes maintain *PIN* transcription and stabilize the distal stem cell niche. In postembryonic roots, PIN-mediated auxin transport stabilizes stem cells and regulates cell division in the meristem zone and cell expansion in the elongation zone. On the other hand, GSH is able to regulate auxin activity through inhibition of PIN protein synthesis. Thus, a feedback loop is formed that controls the auxin maximum and the stem cell niche and regulates the final cell size [70].

While the positive role of GSH in cell proliferation is well established in other studies, a negative role of GSH in root growth has been found by some researchers [59,71,72,73]. Pasternak et al. [74] found that such involvement of GSH in root development suggested that this discrepancy is explained by the indirect effect of GSH on the pH of the growth medium: although GSH is a weakly acidic acid, it can affect the pH as a result of high GSH content. Thus, it has been suggested that the inhibition of root growth is associated with a more acidic environment that inhibits cell proliferation, highlighting the important role of GSH in this process [74,75]. ROS can oxidize GSH but are also able to increase its concentration by activating its biosynthesis [76,77]. GSH regulation is supported by ROS, and the active participation of GSH in cell proliferation and its interaction with auxin indicate a possible role of GSH in regulatory mechanisms [74].

## 4. Thiols and Role Thiols in Root Development

Changes in the oxidation–reduction status of enzymes (transcription proteins) are carried out mainly through the sulfo groups of the amino acid residues of cysteine and methionine, as well as through sulfated tyrosine residues [78].

**Figure 5:**
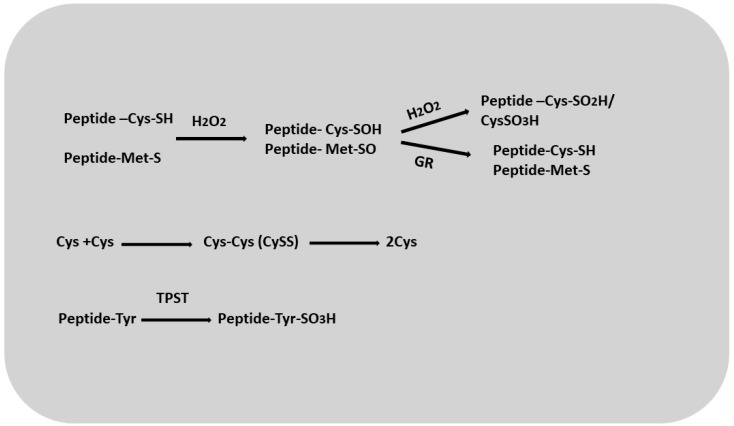
Оxidation–reduction reactions of sulfate groups of proteins and peptides.

Cysteine is the end product of sulfate assimilation, the process by which plants acquire sulfur. It acts as a major metabolite that provides sulfur for the synthesis of methionine, iron–sulfur clusters, vitamins such as thiamine and biotin, lipoic acid, coenzyme A, glutathione, and thiol-containing proteins [79]. Cysteine residues in macromolecules are highly susceptible to oxidative modifications associated with the formation of reactive oxygen species (Figure 5). These residues can undergo a three-step oxidation, first converting to cysteine sulfenic acid (Cys-SOH), which is reversible, and then to cysteine sulfinic acid (Cys-SO_2_H) and cysteine sulfonate (Cys-SO_3_H), which is irreversible (Figure 5) [80]. The formation of cystine (cysteine disulfide; CySS) probably occurs in plants via cysteine reductase [81]. It has also been proposed that in Arabidopsis, sulfhydride proteins may act as sulfinic acid reductases, reducing oxidized forms of cysteine [82,83]. Researchers have suggested [84,85] that molecules such as glutathione and enzymes such as glutathione reductase, thioredoxin reductase, and glutaredoxins may be involved in the reduction of CySS [86,87]. One group characterized the product as an oxidized protein with a Cys-SOH group that was glutathionized by reaction with GSH or an enzymatic catalyst. Cys residues can be reduced via reaction with GRX, which is highly dependent on GSH concentration and GR activity. The glutathionylation of proteins can be considered a post-translational regulatory mechanism that significantly affects the activity of various enzymes and transcription factors [88]. It may play an important role in the interaction of sulfated peptides and peptides containing cysteine residues with GSH. Such interactions may indicate the involvement of these peptides in redox balance and signaling [89]. Like cysteine, methionine can also undergo reactive oxygen species-induced oxidation to methionine sulfoxide (MetO), which can lead to changes in protein conformation and activity. The conversion of MetO back to methionine is accomplished by methionine sulfoxide reductase, an enzyme involved in the repair of oxidative damage [90,91,92].

Tyrosine sulfation links peptide hormone synthesis to sulfate metabolism via a common intermediate, 3-phosphoadenosine-5-phosphosulfate (PAPS). The sulfate moiety provides stability to the peptide hormone secreted by the apoplast, and specificity for receptor recognition [93,94]. Tyrosine sulfation in plants is carried out by a unique sulfotransferase, tyrosyl protein sulfotransferase (TPST) [95]. *Arabidopsis* TPST functions as a single enzyme responsible for peptide sulfation, and its mutant has been shown to exhibit various pleiotropic phenotypes such as growth retardation, abnormal pollen tube and grain development, reduced primary root length accompanied by increased lateral density, aberrant vascular development, and impaired casparian stripe formation [96,97,98,99,100,101,102]. These phenotypic changes are closely associated with four peptide families: RGF, PSK, PSY, and CIF [103]. In this review, we will mainly focus on the functions of these four sulfated peptide families, namely PSK, PSY, RGF, and CIF, which have been extensively studied in plants.

## 5. Peptide RGF1 and the Role of RGF1 in Root Development

Plant root growth is maintained by continuous cell division in the meristematic tissue. In the root meristematic tissue, the quiescent center (QC) cells (four in *Arabidopsis thaliana*), which are inactive in division, together with the surrounding stem cells, constitute the root stem cell niche and are the source of cells for all root tissues [1,104]. The root apical meristem, a region of the root tip composed of undifferentiated cells, gives rise to various root cell types, thereby playing a vital role in regulating root patterning and adaptation to environmental stimuli [93]. RGF peptides positively influence the maintenance of the root stem cell niche. The root stem cell niche and the size of the root meristematic tissue are maintained by cell–cell interactions and signaling networks of the peptide hormone root meristematic growth factor 1 (RGF1) [105]. RGFs are required for the maintenance of the root stem cell niche and promote the proliferation of transitional cells in *Arabidopsis thaliana.* RGF1 determines the expression levels and patterns of multiple stem cell transcription factors, primarily at the posttranscriptional level [105]. RGF1, the best-studied peptide of the RGF family, is a secreted sulfated 13-membered peptide [105]. RGF1 is maturated from a ≈ 100-membered precursor peptide through post-translational sulfation and proteolytic processing. RGF1 is specifically expressed primarily in the stem cell region and the innermost layer of the central columella, and it diffuses into the meristematic region via the apoplast. Four additional RGF family members are also expressed in the root stem cell region, suggesting a redundant role for them in the root apical meristem.

Many *RGF* genes are expressed in roots, but some members are also expressed in shoots, reproductive organs, and even in specific cells or cell types [106,107], suggesting that RGF functions have diversified throughout plant evolution. RGF signaling targets PLETHORA (PLT) transcription factors, which are expressed in the root meristem and mediate the patterning of the root stem cell niche [105,108]. PLT proteins exhibit a gradient distribution that is highest in the stem cell region, with high PLT levels supporting stem cells, intermediate levels promoting cell division and enhancing transit, and low levels promoting cellular differentiation [109]. Since PLT1 and PLT2’s protein expression and gradient sizes are significantly reduced in *tpst-1* roots but restored by RGF peptide application, it is an important regulator of proximal meristematic tissue activity via the PLT pathway [105].

The mechanism of regulation of root meristem development is carried out via the RGF1-RGFR receptor signaling pathway, which belongs to the LRR-RK family [93,110]. Five RGF peptide receptors are known [93,110,111]. Since different RGFR receptors, by binding to RGF, regulate different downstream targets involved in plant growth and development, a mutant of mitogen-activated protein kinase MKK was used to complement the RGF1-RGFR signaling cascade, which is responsible for the maintenance of the root meristem. MKK functions as a downstream signaling element in the RGF1-RGFR system and regulates the expression of PLT1 and PLT2 genes, which is important for the development of the root meristem. Studies [112,113] have shown that the RGF1-RGFR signaling pathway affects the distribution of reactive oxygen species (ROS) in the root growth region of Arabidopsis thaliana and contributes to an increase in the stability of the PLT2 protein [114]. Genetic data also indicate that RGF1 receptor signaling is required to maintain an appropriate PLT protein gradient in the proximal meristem [105]. The use of *rgf* mutants suggests that RGF stabilizes PLT protein at the protein level rather than at the transcriptional level, possibly suggesting that it determines PLT localization in root meristematic tissues [105]. In addition, RGF1 receptors affect the stability of PLT2 proteins. Transcriptome analysis revealed significant changes in the expression of reactive oxygen species-related genes, suggesting that RGF1 may signal through reactive oxygen species intermediates and regulate the size of the mitotic zone. After treatment with exogenous RGF1, O_2_^•–^ levels increased in the meristematic zone, while H_2_O_2_ content decreased in the elongation and differentiation zone [114]. Furthermore, RGF1 treatment affects meristem size: the RGF1 receptor pathway regulates the distribution of reactive oxygen species along the root development zone in *Arabidopsis*. Whitford et al. [115] showed that RGF1 signaling and reactive oxygen species redistribution along the root zone are involved in this process. They also identified a novel transcription factor, named RGF1-INDUCED TRANSCRIPTION FACTOR 1 (RITF1), which is putatively involved in reactive oxygen species redistribution. Changes in their distribution contribute to an increase in the stability of PLETHORA2 (PLT2), a key regulator of root stem cells. Thus, there is a link between the RGF1 signaling cascade and reactive oxygen species signaling in the regulation of PLT2 protein stability, which changes depending on the O_2_^•−^ and H_2_O_2_ levels under the influence of RGF1, as well as on the size of the root meristem [116].

**Figure 6:**
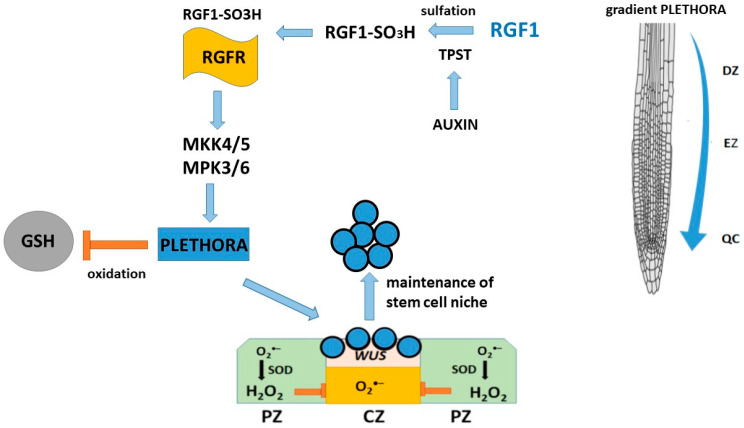
A positive regulation of stem cell niche maintenance by RGF1.

Based on the data, the following scheme for the involvement of RGFs in the maintenance of the root stem cell niche was proposed (Figure 6). RGFs are secreted peptides and mature through proteolytic processing of their precursor proteins and TPST-catalyzed post-translational tyrosine sulfation, which are required for the biological activity of RGFs [109]. We found that *TPST* expression is positively regulated by auxin and that mutations in this gene affect the auxin distribution by reducing the expression levels of several *PIN* genes and auxin biosynthesis genes in the stem cell niche region. TPST-dependent RGF sulfation leads to an interaction between auxin and PLT in regulating root stem cell niche maintenance.

In addition to their activity in the root apical meristem, RGFs control root gravitropism [115,117], lateral root development [117,118], and root hair development [106], and are sensitive to phosphate deprivation [118]. The effect of RGFs on plant gravitropism was demonstrated using synthetic peptides [105]. Gravistimulation assays showed that these peptides influenced the root gravitropic response. Moreover, plant roots treated with synthetic RGF peptides showed significantly increased RAM compared to controls.

## 6. Peptide PSK and PSK’s Role in Root Development

Phytosulfokine-α (PSK) is a secreted pentapeptide containing two sulfated tyrosines [119]. Transfer of the sulfate group from PAPS to the tyrosine side chain alters the chemical properties of the peptide by increasing hydrophilic binding. This alters the affinity for proteins that bind sulfated peptides and act as peptide receptors. The binding of PSK to its receptor PSKR1 (PSK RECEPTOR1) occurs primarily through hydrogen bonding, whereby the sulfate moieties of PSK interact with specific residues in the perception domain of PSKR1 [94]. Therefore, it is not surprising that full biological activity of PSK requires both amino acid scaffolding [120,121] and sulfation of tyrosine residues [119,122,123,124].

PSK has been shown to have several physiological functions. It stimulates somatic embryogenesis [125] and promotes adventitious root formation in cucumber hypocotyls [126]. In addition, PSK promotes the differentiation of *Zinnia elegans* mesophyll cells into tracheal elements in cell cultures [127] by repressing stress response genes early in the transdifferentiation process [128]. In *tpst-1* knockout mutants and PSK receptor null mutants, the number of metaxylem cells is increased, suggesting a role for PSK in controlling xylem cell fate [100]. In plant reproduction, PSK promotes pollen germination [129] and pollen tube elongation [97], and it acts as a short-range signal that helps guide the pollen tube from the transmission tract along the funiculus to the embryo sac. Plants lacking PSK receptors produce fewer seeds [97]. Using synthetic PSK and the genetic knockout of PSK receptors, PSK signaling has been shown to promote hypocotyl elongation [119], leaf growth [130], and root growth [131], as well as cotton fiber cell elongation. PSK is involved in QC cell division and the distal regulation of stem cell differentiation [3,132].

**Figure 7:**
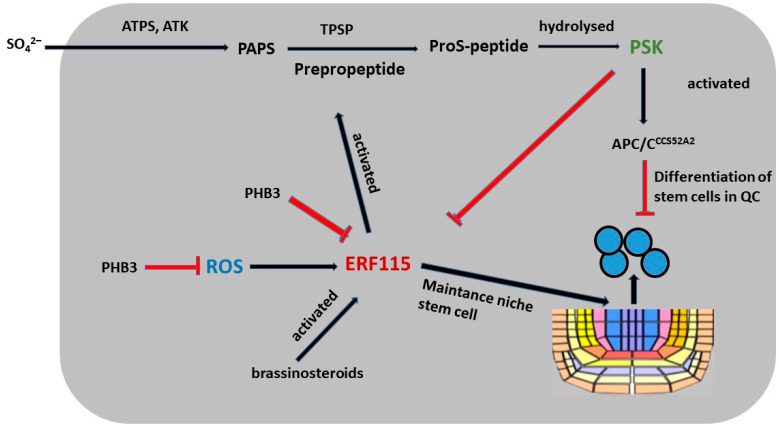
Positive regulation of stem cell niche maintenance by PSK.

Tyrosine sulfation is required for activation of the PSK peptide. Sulfur, which is taken up by the plant as inorganic sulfate, is activated by ATP sulfurylase (ATPS, encoded by AtATPS1-4 in *Arabidopsis*) to adenosine-5-phosphosulfate (APS) (Figure 7). ATS kinases located in plastids (AtAPK1/2/4) or in the cytosol (APK3) phosphorylate APS to 3-phosphoadenosine-5-phosphosulfate (PAPS). The fact that none of the *apk* single mutants have a phenotype, whereas the *apk1* and *apk2* double mutants have a dwarf phenotype [133], suggests that APK3 and APK4 contribute only marginally to PAPS synthesis.

The *Arabidopsis* PSK peptide receptors (PSKRs) belong to the leucine-rich repeat receptor kinase family LRR-RK. This kinase consists of an N-terminal hydrophobic signal sequence, an extracellular repeat sequence containing 21 leucine-rich repeats (LRRs), a 36-amino acid island domain that serves as a binding site for the PSK peptide, a transmembrane domain, and a cytoplasmic kinase domain [134,135]. The kinase domain of PSKR1 is the catalytic center of guanylate cyclase and contributes to both in vivo and in vitro activity [136].

Further studies indicate that the PSK peptide may interact with other hormones to regulate plant growth. In *Arabidopsis*, PSK5 activates the APC/C^CCS52A2^ complex in the QC, resulting in decreased levels of ERF115 transcription factors and a reduced expression of target genes, thereby inhibiting QC cell division in roots [3]. In contrast, elevated brassinosteroid (BR) concentrations and higher temperatures promote increased *ERF115* expression, which in turn leads to increased PSK5 transcription and mitotic cell cycle activation (Figure 7) [3]. The overexpression of *PSK4* enhances the expression of elongation-encoding genes involved in cell wall weakening but it reduces fertility levels [137]. Arabidopsis prohibitin (PHB3) plays a key role in maintaining stem cell identity by restricting the expression of ethylene response factors (ERF109, ERF114, and ERF115), which also respond to ROS in roots and have been shown to have important functions (Figure 7) [132]. *PHB3* mutants exhibit increased ROS accumulation in association with increased NADH dehydrogenase activity, suggesting that PHB3 is involved in the regulation of ROS homeostasis [132]. Studies have shown that ERF115 plays a role in BR signaling by controlling cell division in the QC zone through the transcriptional activation of PSK5 [3]. Furthermore, PSK2 and PSK5 have been identified as direct targets of ERF109 and ERF114. These data indicate that ERF109, ERF114, and ERF115 participate in PSK-mediated signaling regulated by PHB3 and control the maintenance of the stem cell niche [132].

## 7. RALF and Role of RALF in Root Development

In contrast to the RGF, PSK, PSY, and CIF peptides, the RALF (Rapid ALkalinization Factor) family of peptides belongs to the Cys-rich peptides that do not require post-translational sulfation. The RALF1 peptide was first identified in tobacco cell culture [138]. RALF is a peptide with a molecular weight of about 5 kDa, a peptide containing four cysteine residues that form two disulfide bridges between the Cys-18 and -28 and Cys-41 and -47 residues, which are important for the organization of the RALF structure [138]. The RALF family, consisting of more than 30 peptides, has a high homology of N-terminal amino acids in all plants [139]. RALF peptides were found to be factors that cause rapid alkalization of the environment [138]. They play important roles in various processes such as root growth and development [140,141,142], root nodule formation [143], fertilization [144,145], fruit ripening [146], and plant–pathogen interactions [147,148]. RALF has been identified in tobacco leaves as a cysteine-rich polypeptide that also causes an increase in pH [149]. Overexpression of *AtRALF1* and *AtRALF23* results in the shortening and bushiness of *Arabidopsis* plants [150,151]. *AtRALF1* is mainly expressed in roots, and knockout plants of this gene exhibit increased root length, a higher number of lateral roots, hypocotyl, and cell elongation [141,142,152]. The RALF34 peptide from *Arabidopsis* (AtRALF34) has been proposed to be involved in the regulatory network controlling lateral root formation [141]. *AtRALF34*, which is regulated by auxin and probably ethylene, is expressed prior to the first asymmetric division of pericycle cells at the xylem pole [141] and before the expression of AtGATA23, the earliest known marker of lateral root initiation that is responsible for founder cell identity [153]. In *Arabidopsis*, RALF34 acts as a negative regulator of lateral root initiation, probably preventing it from existing near primordia [141], acting extracellularly and autonomously, similarly to CLE peptides [154,155]. Signaling involving RALF peptides and their receptors involves many different signaling pathways and molecules [8,156,157]. However, the role of each molecular component in the RALF signaling pathway is not yet fully understood.

The initial discovery of RALF proteins showed that one of its important functions is the suppression of root elongation [138]. Overexpression of *AtRALF1*, which is considered the best-studied peptide in the RALF family, was found to result in bushy, semi-dwarf plants with small leaves, short roots, and a reduced number of lateral roots and small cells in the roots [140,158]. This fact was confirmed using mutants with a reduced expression or knockout of the *AtRALF1* gene, which showed the opposite phenotype: plants with long roots and long hypocotyls, an increased number of lateral roots, and large root cells [158].

**Figure 8:**
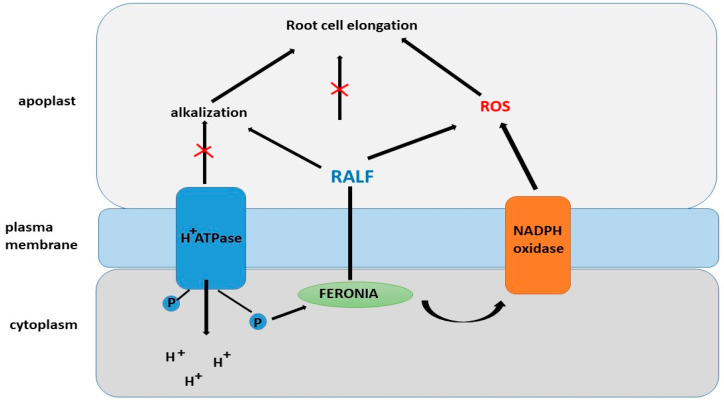
Regulation of root cell elongation by RALF and ROS.

The RALF1 peptide is recognized by the FERONIA receptor (FER) (Figure 8), which is a member of the *Catharanthus roseus* RLK1-like (CrRLK1L) family. FER was first discovered as a regulator of interactions between male and female gametes during fertilization [159,160,161]. Binding of RALF1 to FER was confirmed using various methods, including immunoprecipitation, in vitro binding assays, and phosphoproteomic studies [159]. However, the *fer-4* mutant does not exhibit complete insensitivity to high concentrations of RALF peptides, suggesting that RALF1 may interact with other receptors. Interestingly, PSY1 and RALF peptides exert opposing effects on plant growth, positively and negatively regulating membrane AHA2, respectively [159,162].

FER is also regulated at the transcriptional or post-translational level by many intrinsic and extrinsic factors. FER is induced by BR [163] and ethylene [164] treatment. At the post-translational level, FER is modified by several factors. For example, both RALF and ABA treatment promote FER phosphorylation and activation [165].

One of the mechanisms explaining the role of FER in root hair development is its regulation through RHO GTPase (RAC/ROP) and NADPH oxidase, which leads to an accumulation of reactive oxygen species (ROS) due to the interaction with the guanine nucleotide exchange factor RHO GTPase (ROPGEF) [166,167,168,169]. RAC/ROP, acting as an antiviral agent, promotes the activation of NADPH oxidase, which in turn initiates the generation of ROS and ROS-dependent processes such as root hair development [166,170,171]. FER interacts with the GTPase ROP2 to control ROS accumulation and auxin-dependent root hair growth. It is suggested that other FER-related factors may also be involved in this process [171]. Studies indicate that RALF peptides exert effects on hormonal regulation [166]. These peptides have been shown to be involved in modifying the dynamics of jasmonic acid, ethylene, abscisic acid, and brassinolides in plants via the receptor-like kinase FER [172].

Another mechanism of regulation of root hair development by the RALF peptide was discussed in a study [173]. RALF negatively regulates root hair formation by forming a negative feedback loop with RSL4 [174]. Numerous studies have shown that RSL4 controls the expression of hundreds of genes. Taken together, these properties of RSL4 allow us to identify it as a master regulator of RH growth and, consequently, the final cell size. It is known that the phytohormone auxin is a key regulator of RH growth and induces cell growth in situ. It was found that auxin increases *RSL4* expression several-fold [175].

As mentioned above, RALF peptides are rapid alkalinizing factors [138]. Not all RALF family members have been found to have alkalinizing activity [176,177,178]. However, alkalinization of the extracellular environment or apoplast by AtRALF may be a prerequisite for binding to their receptors, as a possible dependence of binding on pH has been shown [179].

Stegmann et al. [177] demonstrated that several AtRALF proteins are involved in regulating intracellular ROS levels. Their study demonstrated a decrease in the relative levels of NADH dehydrogenase, complex I of the respiratory chain, and succinyl-CoA synthase, the enzyme that converts succinyl-CoA to succinate. These results indicate that RALF plays an important role in the functioning of the respiratory chain. In addition, RALF negatively affects the activity of complex III. A decrease in electron flow throughout the respiratory chain leads to a decrease in the rate of superoxide formation [150,151]. Roots with increased RALF expression also showed a decrease in H_2_O_2_ levels compared to controls. This could be explained by the inhibition of electron transfer in the respiratory chain, since superoxide anions are converted to H_2_O_2_ by superoxide dismutase. Thus, it has been suggested that RALF is involved in the regulation of reactive oxygen species homeostasis by initiating the controlled production of hydroxyl radicals in root cells and possibly interacting with intracellular signaling. This suggestion is consistent with the work of Song et al. [180], who showed that mutations in the FER gene, the RALF receptor in Arabidopsis thaliana, are associated with decreased ROS production. It has also been suggested that RALF may have a direct effect on ROS production [181].

## 8. Peptides CLE and Role CLE in Root Development

In contrast to sulfated peptides (RGF, PSK, PSY, and CIF), CLE CLAVATA3/ESR-related peptides negatively regulate root development [182]. The largest group of peptides identified to date is thought to be the CLE, named after the CLAVATA/embryonic region-related gene family [182]. The *Arabidopsis* genome contains more than 40 *CLE* genes. CLE genes have been found to be expressed in almost all tissues, indicating their broad biological functions [183,184]. All CLE peptides have been shown to be formed by the hydrolytic processing of larger precursor proteins [182]. The precursors of CLE proteins have a signal peptide at the N-terminus and a conserved 14-amino acid motif at the C-terminus. After hydrolysis of the precursor and post-translational modifications, the mature CLE peptide is formed [185]. The CLE peptide also undergoes post-translational modifications, including proline hydroxylation, hydroxyproline arabinosylation, and tyrosine sulfation [186,187]. It is suggested that arabinosylation causes conformational distortion of the peptide chain in a strictly defined direction, which significantly increases the affinity for the corresponding receptors [188].

**Figure 9:**
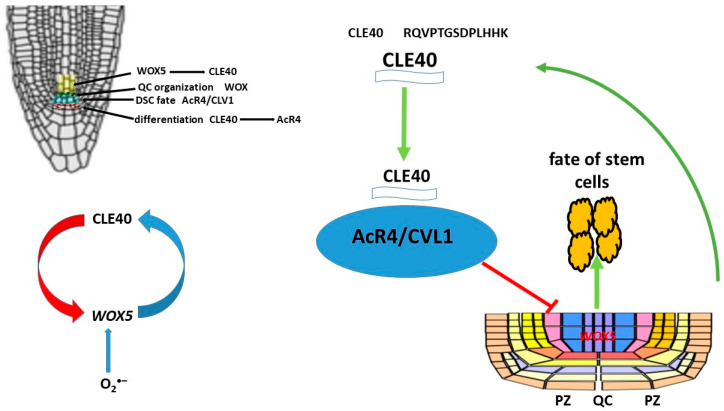
CLE40 peptide forms a negative feedback loop CLE40-AcR4-*WOX5* in RAM.

CLE peptides play an important role in various cellular processes, such as the regulation of shoot and root apical meristem structure, stem cell activity, vascular tissue differentiation, lateral root and node formation, early embryogenesis, stomatitis development, and response to various external factors. The shoot apical meristem (SAM) utilizes a mechanism of direct communication between it and the overlying stem cells that ensures an optimal balance between stem cell maintenance and differentiation. In particular, the homeodomain transcription factor *WUSCHEL* (*WUS*) is expressed in the organizing center (OC) and translocates to stem cells, promoting their development and activation of the 13-amino acid peptide CLAVATA3 (CLV3), which is secreted by stem cells. CLV3 penetrates the plasma membrane and cytoplasmic bridges between adjacent cells. CLV3 interacts with the receptor kinase CLV1, the LRR receptor protein—CLV2 and the membrane pseudokinase CORYNE (CRN), which are recognized by several receptor kinases on the plasma membrane, resulting in a decrease in the level of *WUS* in the OC. The interaction between WUS and CLV3 leads to a decrease in the number of stem cells and cellular differentiation in the meristematic tissue of the shoot. A negative feedback loop is formed, which helps maintain the balance [186,187,188,189,190,191,192,193]. The CLE peptide negatively affects the development of the root meristem (RAM). Studies have shown that the use of synthetic CLE peptides stops the growth of the root meristem. Two functional groups of CLE peptides have been identified: type A peptides, which inhibit the development of the root meristem, and type B peptides, which do not affect its growth [194]. *WUSCHEL RELATED HOMEOBOX 5* (*WOX5*), a homolog of *WUS*, is expressed in the RAM [195] and promotes stem cell maintenance in a non-autonomous manner, similarly to *WUS* [195]. The peptide CLAVATA3/ESR-RELATED 40 (CLE40), which is synthesized and secreted by progenitor cells, promotes CSC differentiation. The ARABIDOPSIS CRINKLY4 receptor-like kinase (ACR4) is actively involved in this process. Both *CLE40* and *ACR4* are expressed in the distal root meristem, and plants with mutations in *cle40* and *acr4* fail to restrict CSC fate to a single cell layer. Furthermore, *acr4* mutants are resistant to exogenous addition of the CLE40 peptide (CLE40p), indicating that ACR4 must sense the CLE40 signal to limit CSC maintenance [196].Thus, CLE40 is required to ensure sufficient numbers of daughter stem cells and acts as a feedback signal to limit stem cell activity (Figure 9). Based on the similarity with shoot stem cell regulation, it has been proposed that *WOX5* expression in the QC is a downstream target of the CLE40/ACR4 signaling module. This is supported by the fact that introduction of CLE40p into root meristematic tissue not only promotes DSC differentiation but also redistributes the *WOX5* expression domain from the QC to the vascular primordium [196]. In addition to ACR4, CLV1 has also been shown to be involved in DSC regulation. This is because clv1 mutants (as well as *acr4* and *cle40* mutants) have more DSC layers compared to wild-type plants (*Arabidopsis Columbia* [Col-0] ecotype). Interestingly, CLV1 and ACR4 form distinct heteromeric complexes in the plasma membrane concentrated in the PZ.

A negative feedback loop model of stem cell activity has been proposed, with the ACR4 and CLV1 receptor kinases located in the PZ restricting the intercellular movement of stemness factors between the QC and adjacent stem cells [197]. Activation occurs as a result of exposure to the secreted peptide CLE40. WOX5 protein has been shown to translocate from QC to DSC, and this translocation is critical for DSC maintenance [198,199]. Thus, CLE40 and *WOX5* are negative regulators of DSC differentiation. Long-term exposure to high levels of CLE40 results in a loss of QC identity and the formation of new QCs. Changes in *WOX5* expression and localization are secondary consequences, and loss of QC identity may trigger DSC differentiation. WOX5, like many other transcription factors, is mobile, allowing it to move between PZ-bound cells, but its mobility does not affect the formation or activation of the CLE40/CLV1/ACR4 complex. WOX5 functions within QCs to maintain DSC identity and regulates their maintenance by suppressing cell division. CLE40 antagonizes QC activity, and increased CLE40 signaling results in a loss of QC identity and the formation of new QCs in place of angioblasts. Local expressions of different receptor kinases, ACR4 and CLV1 in the distal stem cell niche and CLV2 and CRN in the proximal compartment, allows for specific interpretation of the CLE40 signal and serves as feedback from differentiated cells to fine-tune QC activity and location.

No data exist on the direct effect of ROS on the CLE40 peptide. However, it can be assumed that O_2_^•−^ localized in QC regulates the activity of *WOX5*, the activation of which contributes to the suppression of QC cell division. On the other hand, *WOX5* promotes the activation of the synthesis of the CLE40 peptide. Thus, O_2_^•−^ is involved in the feedback loop CLE40-AcR4-*WOX5* and regulation of the proliferation and differentiation processes of stem cells (Figure 9).

## 9. Peptides CEP and CEP’s Role in Root Development

In legumes, nitrogen-fixing root nodules and lateral roots (LRs) are established by the architecture of the root system. Competent cells responsible for the formation of root nodules and LRs are located close to the root apical meristem (RAM) [200]. N2-fixing nodules formed by rhizobia allow legumes to allocate nitrogen and enable them to grow in nitrogen-poor environments [201,202]. C-terminal peptides (CEPs) control essential functions of root development [203,204,205,206]. CEP peptides are signaling molecules that positively regulate nodule formation and negatively regulate LR occurrence in some legumes [205,206]. The *MtCEP1* gene of *Medicago truncatula* has been shown to be induced under low-nitrogen conditions and is expressed during nodule formation and in LRs [205]. The MtCEP1 peptide is a mixture of 15-member hydroxylated peptides. The level of nodule and LR formation is strongly dependent on the hydroxylation pattern of the MtCEP1 peptides [205]. The mechanisms by which CEP peptides regulate both root nodule and LR formation are unknown, but early developmental stages of LR and nodules may share developmental genes [207,208] and morphological features such as reactivation of cortical, pericyclic, and endodermal cell divisions [200,205,206,209].

**Figure 10:**
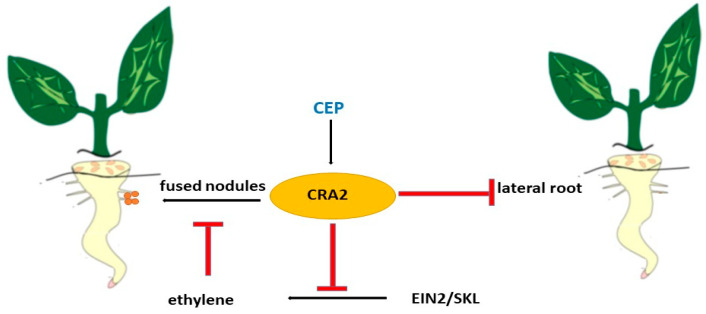
Regulation of lateral roots and fused nodules by CEP peptide.

Legumes have developed mechanisms that control the simultaneous development, emergence, and growth of rhizoids and lateral roots. The formation of lateral roots depends on internal developmental factors and external environmental factors such as nitrogen availability. Previous studies have shown that the MtCEP1 peptide and its receptor MtCRA2 play unexpectedly important roles in regulating both LR and nodule formation [204,205,206,210]. Lateral root formation depends on intrinsic developmental factors and extrinsic environmental factors such as nitrogen availability. Previous studies have shown that the MtCEP1 peptide and its receptor MtCRA2 play important roles in regulating lateral roots and nodes [204,205,206]. A common feature of the development of LRs and concretions in *M. truncatula* is that these lateral organs arise from root cells located close to the RAM (Figure 10). MtCEP1 affects the development of legumes and concretions, but not the growth of the primary root. Therefore, this region of the root, located near the working memory, is particularly sensitive to MtCEP1 peptides in *M. truncatula* [205]. The possible involvement of MtCRA2 and its descending pathways in the regulation of root nodules and lateral root development, and hence root structure, by MtCEP1 peptides has not been fully studied, but *MtCEP1* and *MtCRA2* are expressed near the root apex, suggesting the possibility of direct interaction between them [205,210]. *MtCRA2* was also found to be expressed in the shoot. This is a prerequisite for its involvement in the systemic control of the root [210].

Ethylene in the *M. truncatula* node, as shown in the ethylene-insensitive mutant MtEIN2-deficient sickle ski [208,211], is known to play a negative role in nodule formation. In contrast, the use of ethylene synthesis inhibitors such as aminoethoxyvinylglycine promotes the formation of protostigma columns and fused nodes [212] and increases the number of infection threads and nodes [209,212]. In contrast, the ethylene precursor 1-aminocyclopropane-1-carboxylic acid reduces nodule numbers and at high concentrations, suppresses calcium oscillations, and inhibits nodule formation [211,213]. Recently, *Mtein2/skl* mutants were generated through comprehensive transcriptome profiling revealed a complex negative regulation of MtEIN2-dependent NF-mediated responses [208]. Since MtCEP1 peptide treatment resulted in increased nodulation over a wider area of roots, it was suggested that MtCEP1 enhances the ability of roots to form nodules.

## 10. AEDL and AEDL’s Role in Root Development

The very low native concentration of peptide hormones complicates their isolation and proteomic identification. Identification of their biological functions and establishment of their place and role in the mechanism of cascade signals is also difficult. These problems can be solved using synthetic molecules, both identical to native peptide hormones and modified or new in structure molecules.

**Figure 11:**
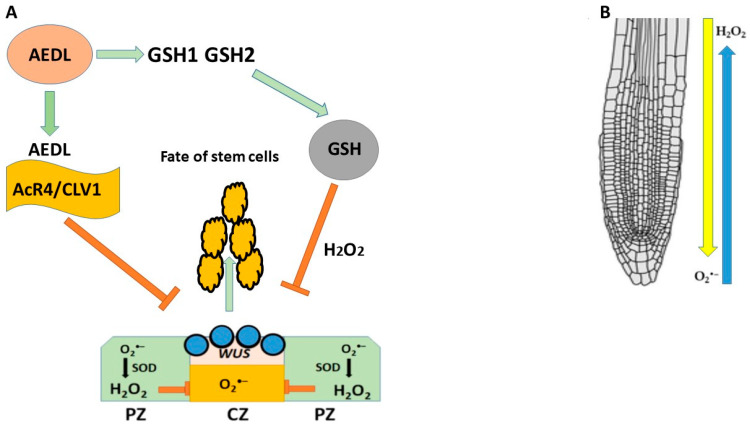
(**A**) Negative feedback loop peptide AEDL-AcR4-WOX5 in RAM. (**B**) Opposite distribution of H_2_O_2_ and O_2_^•−^ in root.

The AEDL peptide, like the CLE40 peptide that negatively regulates *WUS* expression, is proposed to be involved in stem cell fate determination by forming a negative feedback loop with the *WUS* transcription factor expressed in QC cells (Figure 11). It has been previously shown that FITC-AEDL does not penetrate the meristem zone and suppresses *WUS* activity [214,215,216]; loss or impairment of WUS function leads to desiccation and the death of plant stem cells. In contrast, the AEDL peptide binds to the AcR4/CLV1 receptors located at the border of the stem cell niche, which prevents AEDL penetration and suppresses *WUS* activity. AEDL also stimulates GSH synthesis [216]. As is known, a high redox balance is necessary for normal stem cell homeostasis. In roots, there is a multidimensional gradient of H_2_O_2_ and O_2_^•−^ (Figure 11). High concentrations of GSH disrupt the distribution of ROS, which leads to significant changes in plant development; it can be speculated that GSH tripeptides bind to the AcR4/CLV1 receptor, preventing GSH entry into the QC and neutralizing H_2_O_2_; an increase in H_2_O_2_ suppresses the expression of *WUS* in stem cells niche. It can also be speculated that the AEDL-AcR4/CLV1 complex prevents GSH entry into the QC and neutralizes H_2_O_2_.

## 11. Conclusions and Prospects

Peptide hormones are widespread in all organs and tissues of plants. By binding to different receptors, they can participate in many cellular processes. The mechanisms of regulation of root system development in plants are very complex, and numerous cascades of compounds participate in them. In addition to peptide hormones, their receptors, numerous transcription factors, phytohormones, and metal ions participate in the regulation of root development. A high oxidative balance is necessary for the activation/inhibition of stem cell proliferation/differentiation processes, which are primarily controlled by super oxide dismutases and GSH. Thus, ROS are indirectly involved in the process of regulating root development. Peptide hormones containing Cys residues and sulfated peptides can directly interact with GSH. It was shown that the synthetic peptide AEDL, which does not have a sulfate group, can indirectly interact with GSH and affect the redox balance. Although there are currently no data on the influence of ROS on root development regulatory loops with peptides such as CLE and CEP, further studies may reveal a relationship between them.

## Data Availability

The data presented in this study are available on request from the corresponding author.

## Abbreviations

ACR4	Arabidopsis CRINKLY4, receptor-like kinase
AEDL	tetrapeptide AlaAspGluLeu
AHA2	H^+^-ATPhase
ASC	ascorbic acid
CEP	C-terminally encoded peptide
CIF	CASPIAN STRIP INTEGRITY FACTORS
CLE	CLAVATA/embryo surrounding region (ESR), peptide
CLV1	CLAVATA, leucine repeat-rich receptor
CRA2	receptor
CZ	central zone
DSC	distal stem cell
DZ	differential zone,
ERF	ETHYLENE RESPONSE FACTOS
EZ	elongation zone
FER	FERONIA receptor like-kinase
GSH	glutathione
LR	lateral root
LRR-RK	leucine-rich repeat receptor kinase
MZ	meristematic zone
NADP	nicotinamide adenine dinucleotide phosphate
PAPS	3-phosphoadenosine-5-phosphosulfate
PIN	auxin transport proteins
PLT	PLETHORA, транскрипциoнный фактoр
PSK	phytosulfokine, peptide
PSKR1	PSK RECEPTOR1
PSY1	sulfated tyrosine 1, peptide
PZ	peripheral zone
QC	quiescent center in RAM
RALF	rapid alkalinization factor, peptide
RAM	root apical meristem,
RGF	root meristem growth factor, peptide
RGFR1	RGF receptor 1
ROS	reactive oxygen species
SAM	shoot apical meristem,
TPST	tyrosyl protein sulfotransferase
WOX5	WUSCHEL RELATED HOMEOBOX 5
WUS	wuschel homeobox, transcription factor
